# Combined Effect of Marriage and Education on Mortality: A Cross-national Study of Older Japanese and Finnish Men and Women

**DOI:** 10.2188/jea.JE20190061

**Published:** 2020-10-05

**Authors:** Tami Saito, Tuula Oksanen, Kokoro Shirai, Takeo Fujiwara, Jaana Pentti, Jussi Vahtera

**Affiliations:** 1 Department of Social Science, National Center for Geriatrics and Gerontology, Aichi, Japan; 2 Finnish Institute of Occupational Health, Helsinki, Finland; 3 Department of Public Health, Osaka University, Osaka, Japan; 4 Department of Global Health Promotion, Tokyo Medical and Dental University, Tokyo, Japan; 5 Department of Public Health, University of Turku and Turku University Hospital, Turku, Finland

**Keywords:** cross-national comparison, education, marriage, mortality, older adult

## Abstract

**Background:**

While marriage and education help maintain older adults’ health, their joint association with mortality remains unclear. This cross-national study examined the combined effect of marriage and education on the mortality of older Japanese and Finnish adults.

**Methods:**

Data on 22,415 Japanese and 11,993 Finnish adults, aged 65–74 years, were obtained from the Japan Gerontological Evaluation Study of 2010–2012 and the Finnish Public Sector Study of 2008–2009 and 2012–2013. We followed up on respondents’ survival status for 5 years using public records. Marital status, educational level, and other variables in both datasets were harmonized.

**Results:**

The Cox proportional hazards model showed that unmarried men had a higher mortality risk than married men in both countries (hazard ratio [HR] 1.47; 95% confidence interval [CI], 1.21–1.79 for Japanese and HR 1.94; 95% CI, 1.29–2.91 for Finnish); no such difference was observed in women. The highest mortality risk was observed in unmarried men with tertiary education in both Japan (HR 1.85; 95% CI, 1.21–2.83) and Finland (HR 2.21; 95% CI, 1.26–3.89), when adjusted for baseline age, health-related behaviors, and illnesses.

**Conclusions:**

Our findings showed similarity in the combined effect of marriage and education between Japan and Finland, differing from observations in countries with more apparent socioeconomic health disparities. Further studies should examine the reasons for the excessive mortality risk in highly educated, unmarried men in both countries and consider whether selection bias led to underestimation of the true risk in unmarried older adults with lower education.

## INTRODUCTION

Marriage and education are social factors that are known to help maintain the health of older adults.^1^^–^^5^ Marriage can provide health benefits, or “marital protection,” for older adults by providing better social support, control over undesirable health behaviors, or economic security.^6^ Similarly, higher education can help maintain the health of older adults by providing better access to material, human, and social capital.^7^^,^^8^ Studies examining the interrelationships between marriage and socioeconomic status (SES) have shown that individuals with lower SES, including education levels, are less likely to be married; this is called “marital selection”^6^ and could partially explain why they are at excess risk of declining health.^9^ Several studies have also demonstrated that marriage can moderate this risk among individuals with lower SES.^10^^,^^11^ However, most of these findings were obtained from the United States, where SES inequality in health is quite apparent; thus, they may not be representative of other global regions.

In this study, we selected Japan and Finland, because both are developed countries in Asia and Europe, respectively, with relatively small socioeconomic disparity in health.^12^^,^^13^ Such cross-national comparison will better determine the association between health, marriage, and education and better identify the risk groups across countries, because any similarity in the findings could suggest a universality in these relationships and any differences could imply an influence of the sociocultural background or welfare regimes. Japan and Finland have a comparable gross domestic product (GDP) per capita (Japan: USD 42,293; Finland: USD 43,378),^14^ universal healthcare, public long-term care, and a 9-year public education system. They also have comparable longevity (Japan: 83.9 years; Finland: 81.6 years)^15^ and similar leading causes of years of life lost (as of 2017, Japan: ischemic heart disease, stroke, and Alzheimer’s disease; Finland: ischemic heart disease, Alzheimer’s disease, and stroke).^16^ Conversely, the percentage of public spending on tertiary education per GDP is more than three times higher in Finland than in Japan.^17^ This implies that individuals from different backgrounds who achieve higher education may have different health statuses. Furthermore, social relationships, such as marriage, differ between these countries.^18^

Recent studies in Japan and Finland have shown the vulnerability of men with higher SES against the backdrop of recent economic recessions.^19^^,^^20^ Their vulnerability could be exacerbated by a lack of marital protection. Furthermore, Japanese women with higher education are more likely to be single throughout their life,^21^ which could elevate their risk for mortality.^22^ These findings imply that there is a unique interrelationship among SES, marriage, and health in these countries. Previous comparative studies have found similarities and differences in the association between SES and health in Finland and Japan.^23^^–^^26^ However, the differences in the size of the socioeconomic inequality between the two countries vary according to socioeconomic and health variables. In addition, to the best of our knowledge, no study has compared the association of education or marital status with mortality in Japan and Finland.

Until now, few studies have examined the combined effect of education and marriage on mortality in societies wherein socioeconomic health inequality is less apparent than in the United States. In addition, it remains unclear whether such effects are similar in societies with different sociocultural backgrounds. This cross-national study examined the combined effect of marriage and education on the mortality of Japanese and Finnish older men and women.

## METHODS

### Study participants

Datasets were obtained from the Japan Gerontological Evaluation Study (JAGES) and Finnish Public Sector Study (FPS). In this study, we analyzed data from respondents aged from 65 through 74 years, whose data were linked to public records that provided the dates of death for 5 years from the baseline survey, and who provided information regarding marriage and education.

The JAGES primarily examines the social determinants of health in older adults. A total of 169,215 adults, aged 65 years and older, who were not eligible for the Long-term Care Insurance (LTCI) system’s benefits, were selected using random sampling (in 12 larger municipalities) or complete enumeration (in 16 smaller municipalities). A self-administered survey was conducted between August 2010 and January 2012 (effective response rate: 66.3%). For this study, data from 23,403 adults were available. Of these, 754 who did not provide information regarding marriage and/or education were excluded. The final sample for the analysis contained the data of 10,684 men and 11,965 women (Figure 1A).

**Figure 1A.**
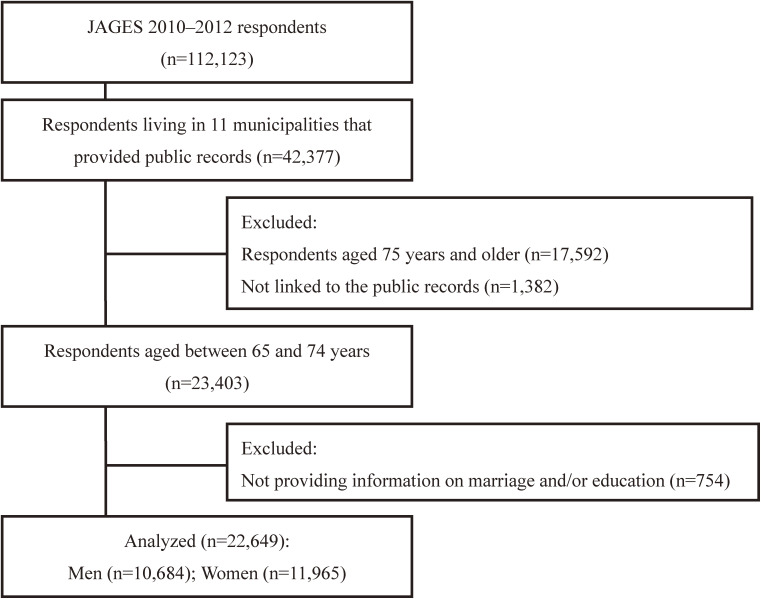
Flowchart of this study (Japanese cohort)

The FPS examines the psychosocial risk factors for ill health and poor functioning across the life span. The FPS includes employees and retirees from a wide range of occupations in 10 towns and six hospital districts. It is followed up by repeat surveys at 4-year intervals and the data are linked to the national health registers. A total of 94,752 employed or retired persons responded to the 2008/2009 and 2012/2013 surveys, with response rates of 64% and 70%, respectively.^27^ For this study, data from those aged 65–74 years (*n* = 12,214) were linked to the Population Register Center for the follow-up of mortality. We excluded those who could not be linked to the Population Register Center (*n* = 97) or who did not provide information regarding marriage and/or education (*n* = 124). The final FPS sample for the analysis included the data of 2,524 men and 9,469 women (Figure 1B).

**Figure 1B.**
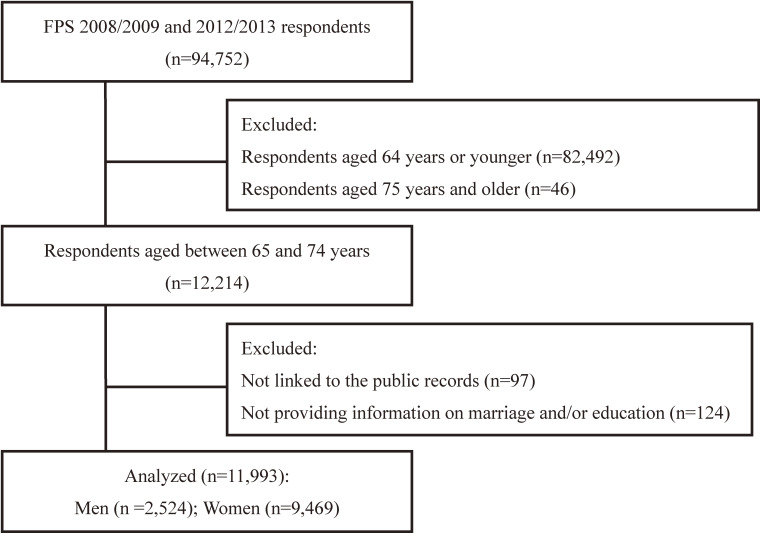
Flowchart of this study (Finnish cohort). JAGES, Japan Gerontological Evaluation Study; FPS, Finnish Public Sector Study.

The study protocol and informed consent procedure of the JAGES were approved by the Nihon Fukushi University Ethics Committee and National Center for Geriatrics and Gerontology. The Ethics Committee of the Hospital District of Helsinki and Uusimaa approved the FPS. Both studies were carried out in accordance with the Declaration of Helsinki.

### Measurements

#### Mortality

From the JAGES, we obtained the all-cause mortality data from the municipality register for deaths that occurred up to 1,827 days following the date when the baseline survey ended, until January 12, 2017. For the Finnish sample (the FPS), data for deaths occurring until December 31, 2016 were obtained from the Population Register Centre of Finland.

#### Marital status and education level

In both cohorts, marital status and education level were self-reported. Marital status was dichotomized (married or cohabitating were coded as married, while widowed, divorced, separated, or never married were coded as unmarried). The level of education was categorized as basic (≤9 years), which was based on the compulsory education systems in both countries, secondary (10 to 12 years), and tertiary (≥13 years). Based on marital status (two categories) and education (three categories), a six-category combination variable for the marriage–education subgroups was created.

#### Baseline covariates

The following health-related behaviors were assessed: smoking (never/former/current), alcohol consumption (no/yes), and body mass index (BMI). For BMI, we categorized <18.5 kg/m^2^ as underweight, between 18.5 and 24.9 kg/m^2^ as normal weight, between 25.0 and 29.9 kg/m^2^ as overweight, and ≥30.0 kg/m^2^ as obese. We assessed the presence of diagnosed chronic illnesses (heart diseases, stroke, diabetes, or cancer) using the link to the health records of the National Drug Reimbursement Register and Finnish Cancer Registry (in the FPS) or using the respondent’s self-report in the baseline survey (in the JAGES).

### Analysis

We employed the Cox proportional hazards model to estimate the hazard ratios (HRs) with 95% confidence intervals (CIs) for mortality. We estimated the age-adjusted HR for marital status and education level. Then, we examined the relative risk for mortality using six combinations of marital status (yes/no) and educational level (basic/secondary/tertiary), while controlling for age (model 1), as well as for the health-related behavior variables and presence of chronic illnesses (model 2). Since the association of marriage or education with mortality can vary by gender or country,^13^^,^^28^ we conducted all analyses by country and gender. We censored the observation at 1,827 days from the baseline. Respondents lost to follow-up due to relocation or other reasons before death were regarded as censored cases (JAGES: 327 cases; FPS: 0 cases). We used IBM SPSS 24.0 (IBM Corp., Armonk, NY, USA) to analyze the JAGES data and SAS v.9.4 (SAS Institute, Marlow, Buckinghamshire, UK) to analyze the FPS data, with the significance level set at *P* < 0.05 (two-tailed).

## RESULTS

In the JAGES, the baseline mean age of both men and women was 69.8 years (standard deviation [SD], 2.6). In the FPS, the baseline mean ages were 68.5 (SD, 2.6) years and 68.3 (SD, 2.5) years for men and women, respectively. During the 5-year follow-up, there were 1,142 deaths in the Japanese cohort, with the mortality rate per 1,000 person-years being 15.4 for men and 6.0 for women. The Finnish cohort had 385 deaths, with mortality rates of 13.8 for men and 7.7 for women (Table 1). The age-adjusted HRs showed that, regardless of country and gender, survivors were more likely to have never smoked and to be free of chronic diseases. When adjusted for age, being unmarried was associated with an increased risk for mortality in Japanese men (HR 1.47; 95% CI, 1.21–1.79) and Finnish men (HR 1.94; 95% CI, 1.29–2.91), but it was only marginally associated with an increased risk for mortality in Japanese women (HR 1.23; 95% CI, 0.99–1.54) and Finnish women (HR 1.27; 95% CI, 1.00–1.63). eTable 1 shows the age-adjusted risk for mortality for marital status of widowed, divorced, and single compared to married. Our results show that Japanese divorced men (HR 1.80; 95% CI, 1.34–2.43) and Finnish divorced men (HR 2.13; 95% CI, 1.30–3.49) had a significantly higher risk for mortality than the married men. Finnish widowed and single women also had a higher risk for mortality (HR 1.48; 95% CI, 1.06–2.05 and HR 1.58; 95% CI. 1.04–2.40, respectively), but such an increased risk was not observed in Japanese women. Education level was related to mortality risk only in Japanese men. Compared to those with basic education, Japanese men with secondary and tertiary education had a reduced risk for mortality (HR 0.81; 95% CI. 0.70–0.95 and HR 0.69; 95% CI. 0.58–0.84, respectively).

**Table 1.** Sample characteristics and age-adjusted hazard ratios for mortality

		Japan				Finland			
		Men		Women		Men		Women	
		(*N* = 10,684; *n* events = 790)		(*N* = 11,965; *n* events = 352)		(*N* = 2,524; *n* events = 125)		(*N* = 9,469; *n* events = 260)	
Variables	Categories	M (SD) or %	HR (95% CI)	M (SD) or %	HR (95% CI)	M (SD) or %	HR (95% CI)	M (SD) or %	HR (95% CI)
Age, 65–74	years	69.8 (2.6)	2.40 (1.83, 3.16)^a^	69.8 (2.6)	2.75 (1.82, 4.17)^a^	68.5 (2.6)	3.13 (0.94, 3.46)^a^	68.3 (2.5)	1.86 (1.18, 2.95)^a^
Marital status	Yes	89.1	1.00	71.4	1.00	85.4	1.0	63.5	1.0
	No	10.9	1.47 (1.21, 1.79)	28.6	1.23 (0.99, 1.54)	14.6	1.94 (1.29, 2.91)	36.5	1.27 (1.00, 1.63)
Education	Basic	36.2	1.00	43.9	1.00	19.5	1.0	18.7	1.0
	Secondary	38.2	0.81 (0.70, 0.95)	40.1	0.91 (0.73, 1.14)	24.3	0.87 (0.53, 1.43)	32.8	0.89 (0.63, 1.23)
	Tertiary	25.6	0.69 (0.58, 0.84)	16.0	0.78 (0.56, 1.08)	56.3	0.69 (0.45, 1.07)	48.5	0.79 (0.58, 1.08)
Smoking	Never	21.8	1.00	80.1	1.00	59.7	1.0	77.6	1.0
	Former	50.4	1.37 (1.12, 1.66)	6.0	2.11 (1.48, 2.99)	31.1	2.29 (1.55, 3.38)	15.2	1.26 (0.89, 1.77)
	Current	21.2	1.75 (1.40, 2.18)	4.0	3.35 (2.37, 4.74)	8.3	3.91 (2.37, 6.46)	6.2	3.01 (2.12, 4.29)
	Missing	6.6	1.38 (1.00, 1.90)	9.9	1.32 (0.94, 1.84)	1.0	1.09 (0.15, 7.91)	1.0	3.42 (1.69, 6.96)
Alcohol consumption	No	35.2	1.00	75.0	1.00	11.9	1.0	24.2	1.0
	Yes	59.5	0.71 (0.61, 0.82)	19.5	0.88 (0.66, 1.16)	87.5	0.65 (0.41, 1.04)	75.1	0.59 (0.46, 0.76)
	Missing	5.3	0.73 (0.52, 1.02)	5.5	0.95 (0.60, 1.52)	0.7	0.89 (0.12, 6.60)	0.7	1.12 (0.36, 3.54)
Body mass index	Underweight	4.1	2.18 (1.69, 2.81)	7.5	1.68 (1.20, 2.34)	0.4	5.63 (1.36, 23.36)	1.1	1.99 (0.81, 4.89)
	Normal	70.6	1.00	68.9	1.00	34.4	1.0	38.6	1.0
	Overweight	22.1	0.81 (0.67, 0.97)	18.4	0.99 (0.74, 1.31)	44.3	0.85 (0.55, 1.32)	37.1	0.82 (0.62, 1.10)
	Obese I	1.7	1.16 (0.69, 1.93)	2.5	1.06 (0.54, 2.06)	18.3	1.73 (1.09, 2.76)	18.1	0.97 (0.69, 1.37)
	Missing	1.6	1.35 (0.83, 2.19)	2.7	1.76 (1.06, 2.92)	2.7	3.03 (1.41, 6.49)	5.2	1.85 (1.19, 2.89)
Any disease	No	68.2	1.00	78.7	1.00	76.9	1.0	87.6	1.0
	Yes	29.8	1.94 (1.68, 2.23)	18.5	2.73 (2.20, 3.40)	23.1	1.99 (1.38, 2.86)	12.4	2.52 (1.91, 3.32)
	Missing	2.0	1.28 (0.78, 2.11)	2.8	1.35 (0.72, 2.55)	0.0	—	0.0	—

CI, confidence interval; HR, age-adjusted hazard ratio; SD, standard deviation.

^a^Per 10 years.

Figure 2A, Figure 2B, Figure 2C, and Figure 2D show the survival curve for each subgroup, according to marital status and education level of the Japanese and Finnish men and women within model 1. Among Japanese men and women, differences in mortality based on educational level graphically appeared to be larger in the married than unmarried groups, while such typical differences were not observed in the Finnish men and women.

**Figure 2A.**
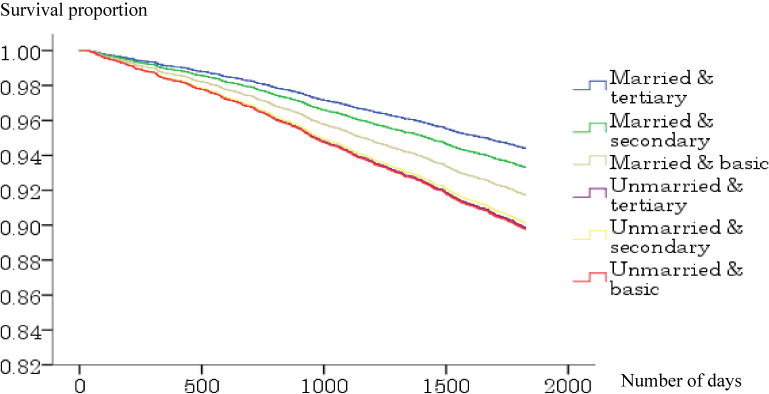
Survival curve for the six groups based on marital and educational status (Japanese men). Adjusted for age.

**Figure 2B.**
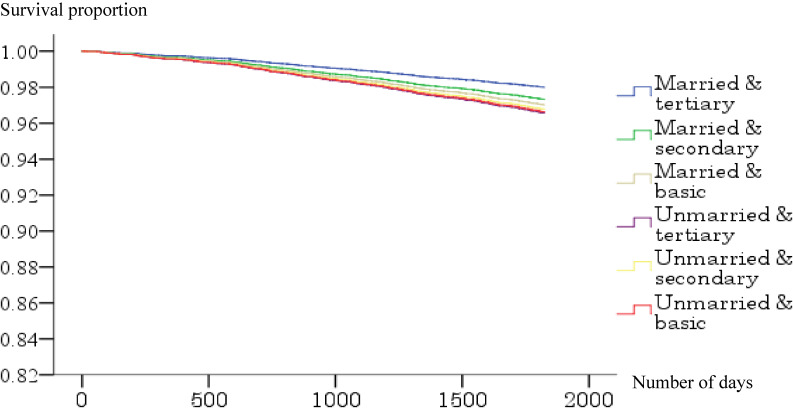
Survival curve for the six groups based on marital and educational status (Japanese women). Adjusted for age.

**Figure 2C.**
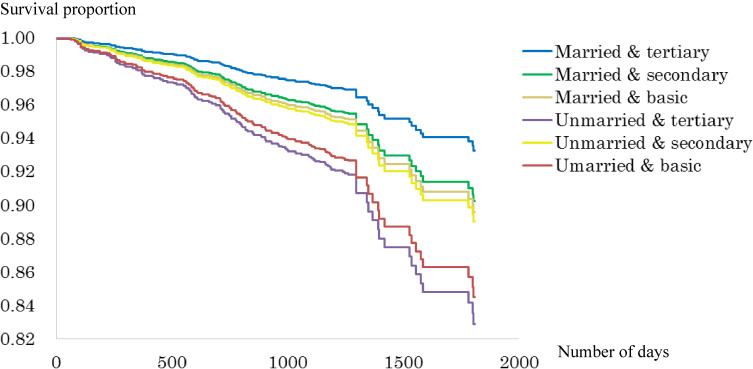
Survival curve for the six groups based on marital and educational status (Finnish men). Adjusted for age.

**Figure 2D.**
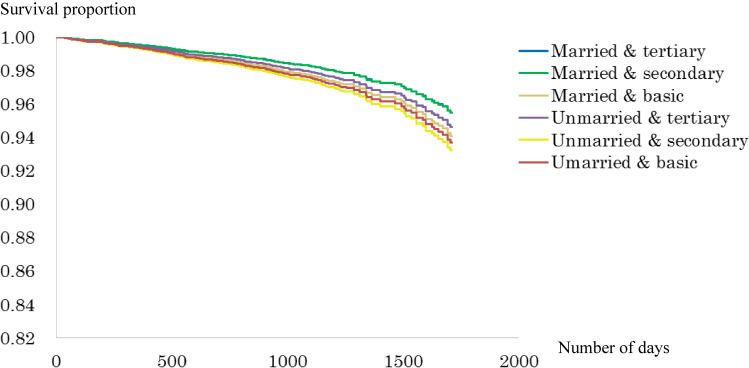
Survival curve for the six groups based on marital and educational status (Finnish women). Adjusted for age.

Table 2 shows the results of the association of the six combinations of marital status and education level by gender and country, using married men and women with tertiary education as the reference group for having both possible protective factors. The Japanese men in all of the subgroups, except for married men with secondary education, had a statistically significantly higher risk for mortality. The highest point estimate was observed in unmarried men with tertiary education (HR 1.85; 95% CI. 1.21–2.83), followed by married men with basic education (HR 1.72; 95% CI. 1.25–2.36), unmarried men with secondary education (HR 1.61; 95% CI, 1.13–2.30), and married men with basic education (HR 1.44; 95% CI, 1.17–1.76) in the fully adjusted model. No excess risk was found for Japanese women in any of the subgroups. Among Finnish men, only those who were unmarried with tertiary education had a significantly higher risk for mortality compared to married men with tertiary education (HR 2.21; 95% CI, 1.26–3.89 in the fully adjusted model). Among Finnish women, no significant difference in the risk for mortality was found in any of the subgroups.

**Table 2.** Hazard ratios for mortality in the six sub-groups by marriage and education categories

**Japan**	Men			Women		
		Model 1^a^	Model 2^a^		Model 1^a^	Model 2^a^
	*n*	HR (95% CI)^b^	HR (95% CI)^b^	*n*	HR (95% CI)^b^	HR (95% CI)^b^
Married & tertiary	2,490	1.00	1.00	1,372	1.00	1.00
Married & secondary	3,683	1.20 (0.98, 1.48)	1.16 (0.94, 1.42)	3,540	1.34 (0.87, 2.06)	1.32 (0.86, 2.03)
Married & basic	3,350	1.50 (1.23, 1.84)	1.44 (1.17, 1.76)	3,631	1.50 (0.98, 2.28)	1.43 (0.94, 2.18)
Unmarried & tertiary	246	1.86 (1.21, 2.84)	1.85 (1.21, 2.83)	543	1.73 (0.96, 3.11)	1.54 (0.86, 2.78)
Unmarried & secondary	402	1.81 (1.27, 2.58)	1.61 (1.13, 2.30)	1,263	1.62 (1.00, 2.62)	1.47 (0.91, 2.39)
Unmarried & basic	513	1.88 (1.37, 2.57)	1.72 (1.25, 2.36)	1,616	1.69 (1.07, 2.68)	1.43 (0.90, 2.27)

**Finland**	Men			Women		
		Model 1^a^	Model 2^a^		Model 1^a^	Model 2^a^
	*N*	HR (95% CI)	HR (95% CI)	*N*	HR (95% CI)	HR (95% CI)

Married & tertiary	1,240	1.00	1.00	3,028	1.00	1.00
Married & secondary	510	1.47 (0.90, 2.40)	1.22 (0.74, 2.01)	1,939	1.00 (0.69, 1.46)	0.92 (0.63, 1.35)
Married & basic	405	1.58 (0.96, 2.61)	1.25 (0.75, 2.08)	1,050	1.32 (0.88, 2.00)	1.14 (0.75, 1.73)
Unmarried & tertiary	180	2.70 (1.55, 4.70)	2.21 (1.26, 3.89)	1,565	1.20 (0.82, 1.75)	1.09 (0.75, 1.60)
Unmarried & secondary	103	1.67 (0.71, 3.92)	1.17 (0.49, 2.78)	1,164	1.52 (1.04, 2.24)	1.17 (0.79, 1.74)
Unmarried & basic	86	2.41 (1.14, 5.11)	2.03 (0.94, 4.37)	723	1.41 (0.90, 2.22)	1.06 (0.67, 1.68)

CI, confidence interval; HR, hazard ratio.

^a^Model 1 adjusted for age; model 2 additionally adjusted for smoking, alcohol consumption, BMI, and having any chronic disease.

## DISCUSSION

This large cross-national study examined the combined effect of education and marriage on the mortality of Japanese and Finnish older adults. In both countries, unmarried men with tertiary education had the highest risk for mortality. This suggests that the excess risk for mortality in highly educated and unmarried men may be common across different sociocultural backgrounds, at least among Japanese and Finnish older adults. The reason for this excess risk remains unclear because it was still observed when adjusting for the presence of chronic illnesses and behavior-related health risks, such as smoking, high alcohol intake, and obesity. Although not assessed in this study, other factors, such as a lack of social support from a spouse, another component of marital protection,^6^ or less resilience, may play a role in the increased risk.

These findings do not support those of previous studies conducted in the United States,^11^^,^^29^^,^^30^ because our findings show neither a cumulative nor moderating effect of marriage and education on mortality. This discrepancy could be partially explained by smaller differences in the mortality risk across educational levels in Japan and Finland than in the United States.^13^^,^^31^ Therefore, it is possible that the combined effect of education level and marital status on mortality may depend on the level of socioeconomic inequalities in health within countries.

However, because our study targeted adults aged at least 65 years, those with the highest mortality risk may have died before that age. Such a selection bias would lead to an underestimation of the mortality risk in unmarried individuals with a lower education level. Indeed, in our study, the relatively low risk for mortality in unmarried Finns without tertiary education suggests such a possibility, because educational inequalities in health are apparent in Japan and Finland.^3^^,^^31^^,^^32^

In addition, our findings showed a gender difference, with a stronger association between marriage and mortality in men than women in both countries. Other studies have found similar gender differences,^28^ suggesting that husbands rely more on social support exchange with their wives,^33^ who also take a larger role in controlling their husbands’ health-related behaviors.^34^ In particular, our additional analysis found a significantly higher risk in divorced men as compared to married men in both countries. Divorced men had higher risk for suicidal,^35^ accidental, violent, or alcohol-related death.^36^ Although our data did not include information on detailed causes of death or risk behaviors, such as binge drinking, more attention should be paid to the risks of divorced men in both countries.

Although these cross-national data provide unique findings, we should note several limitations. Because we measured the health-related behaviors and presence of illnesses only at the baseline, we do not know how changes in health and health risks could have affected the observed associations of education and marriage with mortality. Furthermore, causal relationships between marital status and health or health-related variables remain unclear. Although we used the data of relatively healthy older adults and controlled for health-related behaviors and health variables, it is still possible that several unmeasured confounders, such as undiagnosed medical conditions, may bias the relationship of marriage and education with mortality. Additionally, the JAGES and FPS cohort samples vary regarding sample selection, assessment methods, and mean age, even though the study variables were harmonized. For instance, the JAGES collected data from functionally independent older adults, while the FPS data were obtained from public sector employees and retirees. These differences may have affected the comparability or generalizability of the findings.

In conclusion, this cross-national study showed quite similar findings regarding the association among education, marriage, and mortality in Japan and Finland, despite the differences in sociocultural backgrounds and welfare regimes. Our findings suggest a possibility that the combined effect of marriage and education on mortality varies according to the socioeconomic health inequality within countries. Further studies should elaborate on the possibility of using data from more diverse countries. In addition, professionals working with older adults should pay more attention to the combination of social circumstances in older adults. We uncovered a vulnerability among unmarried highly educated men in both countries, whose risk tends to be overlooked. Further studies should identify the mechanisms in order to explain the association between being unmarried with tertiary education and mortality risk. There may be several pathways (lack of social support, health information, resilience, or other reasons) for such mechanisms. At the same time, we should consider the possibility of selection bias that underestimates the mortality risk in unmarried individuals with a lower education level.

## APPENDIX A. SUPPLEMENTARY DATA

eTable 1. Age-adjusted hazard ratios for mortality of marital status sub-groups
