# Supplementary material for: Combined Effect of Marriage and Education on Mortality: A Cross-national Study of Older Japanese and Finnish Men and Women
Source: J Epidemiol. 2020 Oct 5;30(10):442–9. doi: 10.2188/jea.JE20190061 (PMC7492707; doi:10.2188/jea.JE20190061)
Supplement: Supplementary file 1 [file je-30-442-s001.pdf]

eTable 1. Age-adjusted hazard ratios for mortality of marital status sub-groups

		Japan				Finland			
		Men (N=10,684; n events=790)		Women (N=11,965; n events=352)		Men (N=2,524; n events=125)		Women (N=9,469; n events=260)	
Variables	Categories	M (SD) or %	HR (95% CI)[a]	M (SD) or %	HR (95% CI)[a]	M (SD) or %	HR (95% CI)[a]	M (SD) or %	HR (95% CI)[a]
Marital status	Married	89.1	1.00	71.4	1.00	85.4	1.0	63.5	1.0
	Widowed	5.0	1.32 (0.99, 1.75)	21.4	1.17 (0.91, 1.50)	3.3	1.53 (0.67, 3.50)	13.1	1.48 (1.06, 2.05)
	Divorced	3.6	1.80 (1.34, 2.43)	4.9	1.35 (0.87, 2.12)	8.3	2.13 (1.30, 3.49)	16.5	0.98 (0.69, 1.40)
	Single	2.2	1.31 (0.84, 2.05)	2.3	1.58 (0.88, 2.82)	3.0	1.89 (0.83, 4.32)	6.8	1.58 (1.04, 2.40)

CI, confidence interval; HR, age-adjusted hazard ratio; SD, standard deviation.
